# Smoking prevalence trends in Indigenous Australians, 1994-2004: a typical rather than an exceptional epidemic

**DOI:** 10.1186/1475-9276-8-37

**Published:** 2009-10-31

**Authors:** David P Thomas

**Affiliations:** 1 Menzies School of Health Research, PO Box 41096, Casuarina, NT, 0811, Australia; 2 Institute of Advanced Studies, Charles Darwin University, NT, 0909, Australia; 3 Centre for Health and Society, University of Melbourne, Victoria, 3010, Australia

## Abstract

**Background:**

In Australia, national smoking prevalence has successfully fallen below 20%, but remains about 50% amongst Indigenous Australians. Australian Indigenous tobacco control is framed by the idea that nothing has worked and a sense of either despondency or the difficulty of the challenge.

**Methods:**

This paper examines the trends in smoking prevalence of Australian Indigenous men and women aged 18 and over in three large national cross-sectional surveys in 1994, 2002 and 2004.

**Results:**

From 1994 to 2004, Indigenous smoking prevalence fell by 5.5% and 3.5% in non-remote and remote men, and by 1.9% in non-remote women. In contrast, Indigenous smoking prevalence rose by 5.7% in remote women from 1994 to 2002, before falling by 0.8% between 2002 and 2004. Male and female Indigenous smoking prevalences in non-remote Australia fell in parallel with those in the total Australian population. The different Indigenous smoking prevalence trends in remote and non-remote Australia can be plausibly explained by the typical characteristics of national tobacco epidemic curves, with remote Indigenous Australia just at an earlier point in the epidemic.

**Conclusion:**

Reducing Indigenous smoking need not be considered exceptionally difficult. Inequities in the distribution of smoking related-deaths and illness may be reduced by increasing the exposure and access of Indigenous Australians, and other disadvantaged groups with high smoking prevalence, to proven tobacco control strategies.

## Introduction

Australia has successfully reduced national daily smoking prevalence (aged 14 and over) to 16.6% [1], and national tobacco control policy debate is now concentrated on how to reduce national smoking prevalence to 9% by 2020 [2]. In contrast, the most recent national survey of Indigenous Australians reported that 50% aged 18 and over were daily smokers [3]. There are two distinct groups of Indigenous peoples in Australia: Aboriginal peoples and Torres Strait Islanders. One-quarter of Indigenous Australians (compared with 2% of the total Australian population) live in remote areas with less access to most services. Nevertheless Indigenous Australians live in all the parts of the country, including all states and territories, and comprise 2.5% of the Australian population (about 90% of whom are Aboriginal people) but have much poorer health than other Australians [4]. A public campaign to reduce this health inequality with the slogan 'Close the Gap' was launched in 2007 by Indigenous, human rights and other non-government organizations [5]. The language of the campaign was adopted by the Australian Labor Party, first in Opposition and then in government following the 2007 Australian Federal election.

Reducing Aboriginal and Torres Strait Islander smoking and smoking-related harm is a central element in the Australian government's efforts to improve Indigenous health and to 'Close the Gap' between Indigenous and other Australians. Smoking is estimated to cause 20% of Indigenous deaths, and to be responsible for 17% of the health gap between Indigenous and other Australians [6,7]. Smoking is the largest single risk factor contributing to the Indigenous disease burden and the health gap with other Australians. In March 2008, Australian Prime Minister Rudd announced that his government would spend an additional $14.5 million over four years on Indigenous tobacco control as one of his government's first steps to 'Close the Gap' [8]. Later that year, further funds for tackling Indigenous smoking were announced as part of the $1.6 billion investment by the Council of Australian Governments in 'Closing the gap' [9].

Whilst Australian tobacco control advocates struggle with reduced media and public interest due to the perception that all their battles have been won [10], Australian Indigenous tobacco control is framed by the idea that nothing has worked and a sense of either despondency or the difficulty of the challenge. Several recent government and research reports comment that the prevalence of smoking in the Australian Indigenous population has not changed, whilst smoking has fallen in the Australian population [4,11-14], These comments are mainly based on the estimates of total Indigenous smoking prevalence in three national Indigenous surveys performed by the Australian Bureau of Statistics (ABS) in 1994, 2002 and 2004: the National Aboriginal Torres Strait Islander Survey (NATSIS), Social Survey (NATSISS), and Health Survey (NATSIHS). Some, however, also use the smaller Indigenous samples in the 1995 and 2001 National Health Surveys. However, as is only rarely noted [15], the different survey reports used different age cut-offs or different definitions of smoking. Researchers have not performed the detailed examination of Indigenous smoking prevalence trends as has occurred for the total Australian population [16]. A notable exception is the recent paper describing falling smoking prevalence from 1996 to 2005 in both Indigenous and non-Indigenous students aged 12 to 17 years participating in triennial surveys of secondary school students [17].

This paper examines the trends in smoking prevalence of Indigenous men and women aged 18 and over in three large national surveys in 1994, 2002 and 2004, and the implications for tobacco control in this high smoking prevalence disadvantaged group. The next national Indigenous survey has been completed, however, results will not be available until 2010.

## Methods

The National Aboriginal Torres Strait Islander Survey was conducted from April to July 1994, the National Aboriginal Torres Strait Islander Social Survey from August 2002 to April 2003, and the National Aboriginal Torres Strait Islander Health Survey from August 2004 to July 2005 [3,18,19]. All three surveys used multi-stage sampling strategies. The 1994 NATSIS sampled all residents from randomly selected households from a stratified sample of Census Collection Districts. The 2002 NATSISS and 2004 NATSIHS randomly sampled up to three residents from randomly selected households from either a stratified sample of Census Collection Districts or a random sample of discrete Indigenous communities and outstations. Non-private dwellings (e.g. hostels, hospitals, caravan parks and prisons) were only sampled in the first survey, but these were excluded from the file analysed in this paper [20]. Response rates were about 80-90% in each survey [3,19,21].

Analyses used STATA Version 10 with the Confidentialised Unit Record Files (CURF) for each survey via ABS's Remote Data Laboratory. Under this arrangement, researchers do not have direct access to the datasets, but instead submit statistical code to ABS, which runs the commands and returns the results to the researchers. All analyses used the expansion factor (or person weight) for each respondent to adjust for the disproportionate sampling of some groups, and so estimates reflect the total Indigenous population not just the sample [22]. These weights are based on the Indigenous estimated resident population in private dwellings on 30 June 1994, 31 December 2002 and 2004 [3,19,20]. Confidence intervals were calculated using the replicate weights for each person generated by ABS [22]. As ABS only created 100 replicate weights for the first survey but 250 for the subsequent surveys, it was not possible to combine the files to directly compare smoking prevalences between surveys (and estimate the confidence intervals of any differences) or to build logistic regression models to examine trends in more detail.

The surveys differed in the ways that smoking status was determined and other characteristics of respondents were classified. The first survey only reported whether people smoked or did not; the second survey asked if current smokers were daily smokers or not; the third survey asked if current smokers were either daily, at least weekly or less than weekly smokers. So even though the reports based on the last two surveys describe prevalences of daily smokers, this paper concentrates on prevalences of current smokers: the only consistent category. The last survey only asked people aged 18 and over about smoking, so this paper concentrates on this age group even though younger people were asked the question in earlier surveys. Variables for state or territory of Australia were not included in the CURF for the first survey, so the analyses using state or territory for this survey were performed separately by ABS using the original dataset. Remote regions include those classified by the Accessibility/Remoteness Index of Australia (ARIA) as remote or very remote and include most of the continent: all of the arid inland and almost all of the tropical North. Indigenous status was only available for those in Queensland in the last two surveys (62% of Torres Strait Islanders lived in Queensland in 2006) [4]; in the last survey if respondents were both Torres Strait Islander and Aboriginal they were classified as Torres Strait Islander, so I treated those in the 2002 survey similarly even though a separate category for both Aboriginal and Torres Strait Islander was available. In the first survey, those who listed both Indigenous groups, were classified as Aboriginal if they listed Aboriginal before Torres Strait Islander, and vice versa.

There were 7,710 and 8,523 and 5,757 Indigenous people aged 18 and over who responded to the 1994, 2002 and 2004 surveys. Data on smoking status was missing for ten, seventy and one respondents aged 18 and over in the three surveys. These non-respondents were excluded in all calculations of smoking prevalence.

Ethical approval was given by the Human Research Ethics Committee of the NT Department of Health and Families and Menzies School of Health Research, including its Aboriginal subcommittee.

## Results

Smoking prevalence in the Australian Indigenous population 18 and over declined by 2.4% from 1994 to 2004: from 54.5% (95% CI 51.7-57.4) in 1994 to 53.5(CI 51.0-56.0) in 2002 to 52.1% (CI 49.9-54.3) in 2004.

**Figure 1:**
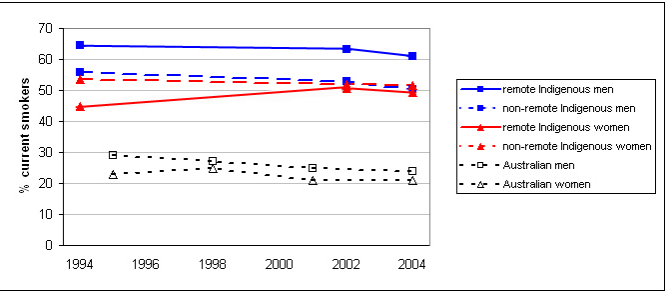
**Prevalence of smoking among Australians aged 18 and over, 1994 to 2004-Indigenous men and women in remote and non-remote areas compared with all Australian men, all Australian women**. Sources: Weighted data from National Aboriginal Torres Strait Islander Survey 1994, the National Aboriginal Torres Strait Islander Social Survey 2002 and the National Aboriginal Torres Strait Islander Health Survey 2004; and Winstanley and White (2008).

Figure 1 shows different smoking prevalences and trends for men compared to women and for remote and non-remote Australia (and the corresponding total Australian smoking prevalence trends)[16]. Most differences are small, with overlapping confidence intervals suggesting that differences may have occurred by chance, but some consistent trends are apparent (see Tables 1 and 2). There were much larger differences between the smoking prevalences of men and women in remote regions, which decreased from 1994 to 2004, than between men and women in non-remote regions. In each survey, men in remote regions had the highest smoking prevalence and women in remote regions the lowest; men and women from non-remote regions had similar smoking prevalences between these extremes. From 1994 to 2004, smoking prevalence fell by 5.5% and 3.5% in non-remote and remote men, and by 1.9% in non-remote women. In contrast, smoking prevalence rose by 5.7% in remote women from 1994 to 2002, before falling by 0.8% between 2002 and 2004.

**Table 1:** Percentages of Indigenous men aged 18 and over who smoked in each survey

	**1994**	**2002**	**2004**
**Age (years)**			
18-24	58.5 (51.1-65.9)	58.3 (50.5-66.1)	53.6 (47.0-60.3)
25-34	65.5 (58.7-72.2)	58.1 (52.4-63.7)	57.3 (50.3-64.2)
35-44	60.1 (54.0-66.2)	60.5 (54.2-66.8)	59.2 (54.1-64.3)
45-54	52.8 (45.5-60.0)	52.1 (45.1-59.1)	51.5 (43.6-59.5)
55+	42.4 (28.0-56.8)	41.6 (32.8-50.3)	36.2 (28.8-43.5)
**Region**			
Remote	64.5 (60.9-68.1)	63.3 (60.0-66.6)	61.0 (56.4-65.7)
Non-remote	55.8 (50.8-60.7)	52.8 (48.7-56.8)	50.3 (46.2-54.5)
**Jurisdiction**			
New South Wales	56.3 (47.5-65.1)	53.0 (45.9-60.1)	52.8 (45.6-60.1)
Victoria	54.4 (40.1-68.7)	52.1 (44.0-60.2)	57.4 (44.1-70.7)
Queensland	57.7 (50.4-65.0)	58.5 (52.3-64.8)	52.7 (46.5-58.9)
South Australia	64.7 (54.9-74.5)	51.5 (44.5-58.5)	59.1 (50.3-67.9)
Western Australia	56.9 (51.4-62.4)	52.2 (45.6-58.9)	43.5 (36.4-50.6)
Northern Territory	66.2 (60.5-71.9)	65.6 (59.9-71.2)	64.4 (57.1-71.8)
Tasmania/ACT	55.8 (46.8-64.8)	50.2 (43.9-56.5)	46.4 (38.7-54.1)
Indigenous status (Queensland only)*			
Torres Strait Islander	48.5 (35.0-62.0)	50.4 (41.7-59.1)	45.9 (35.0-56.8)
Aboriginal	59.6 (51.4-67.8)	59.8 (52.0-67.5)	54.6 (47.6-61.6)

Sources: Weighted data from National Aboriginal Torres Strait Islander Survey 1994, the National Aboriginal Torres Strait Islander Social Survey 2002 and the National Aboriginal Torres Strait Islander Health Survey 2004.

* Torres Strait Islanders in Queensland who also identified as Aboriginal were classified as Torres Strait Islander in 2002 and 2004, but slightly differently in 1994.

**Table 2:** Percentages of Indigenous women aged 18 and over who smoked in each survey

	**1994**	**2002**	**2004**
**Age (years)**			
18-24	52.6 (46.5-58.8)	56.9 (50.0-63.8)	51.6 (44.7-58.5)
25-34	60.4 (55.5-65.4)	57.2 (52.8-61.7)	55.0 (49.6-60.3)
35-44	53.2 (46.0-60.4)	55.0 (50.0-60.0)	58.7 (53.2-64.2)
45-54	45.7 (38.2-53.1)	46.4 (39.5-53.3)	52.3 (45.7-58.8)
55+	24.4 (15.5-33.3)	30.2 (23.9-36.5)	26.7 (20.1-33.3)
**Region**			
Remote	45.0 (40.7-49.4)	50.7 (46.4-55.0)	49.5 (44.8-54.2)
Non-remote	53.5 (49.5-57.6)	51.8 (48.2-55.3)	51.6 (47.9-55.3)
**Jurisdiction**			
New South Wales	56.3 (49.8-62.8)	54.7 (48.5-60.9)	54.1 (47.1-61.2)
Victoria	65.4 (53.1-77.7)	59.5 (53.4-65.6)	46.9 (35.4-58.4)
Queensland	49.8 (43.9-55.7)	49.5 (44.2-54.8)	49.0 (43.7-54.3)
South Australia	55.9 (45.5-66.3)	48.4 (40.5-56.4)	52.7 (45.8-59.7)
Western Australia	48.9 (41.8-56.0)	48.3 (41.3-55.2)	51.8 (45.5-58.1)
Northern Territory	39.1 (32.8-45.4)	51.5 (44.4-58.5)	47.8 (40.0-55.5)
Tasmania/ACT	44.4 (33.8-55.0)	45.3 (40.3-50.4)	53.1 (46.0-60.1)
Indigenous status (Queensland only)*			
Torres Strait Islander	39.7 (27.4-52.0)	50.5 (41.0-60.1)	45.9 (36.8-55.0)
Aboriginal	52.0 (45.9-58.1)	49.0 (42.7-55.2)	49.8 (43.7-55.9)

Sources: Weighted data from National Aboriginal Torres Strait Islander Survey 1994, the National Aboriginal Torres Strait Islander Social Survey 2002 and the National Aboriginal Torres Strait Islander Health Survey 2004.

* Torres Strait Islanders in Queensland who also identified as Aboriginal were classified as Torres Strait Islander in 2002 and 2004, but slightly differently in 1994.

In New South Wales, the Australian jurisdiction with the largest Indigenous population, there were small consistent falls of 3.5% and 2.2% in smoking prevalence in men and women, from 1994 to 2004. In Western Australia, smoking prevalence in men fell by a large 13.4% from 1994 to 2004. In contrast, smoking prevalence in women in the Northern Territory rose by 12.4% from 1994 to 2002, before falling slightly by 2004. In Queensland, Torres Strait Islander men and women had consistently lower smoking prevalences than Aborigines in the surveys.

## Discussion

The lack of comparability between national Indigenous survey results has seriously limited the ability of Australian tobacco control advocates and policy makers to accurately assess progress in reducing Indigenous smoking. By reanalysing the surveys using standardised classifications of smoking status and age, this study goes some way to rectifying this deficiency.

While the insufficient power of the surveys to more precisely measure smoking prevalence, and so identify small changes between surveys, suggests caution in the interpretation of the results, this study indicates that it may not be true that Indigenous smoking prevalences have remained largely unchanged whilst Australian smoking prevalences have fallen, as has conventionally been stated by many in the past. Australian smoking prevalences in men and women aged 18 and over fell by 5% (29 to 24%) and 2% (23 to 21%) from 1995 to 2004 [16]. Almost all (98%) of the non-Indigenous population live in non-remote regions [4]. Male and female Indigenous smoking prevalences in non-remote Australia fell by 5.5% and 1.9% in parallel with these total Australian smoking prevalences, albeit from a much higher initial prevalence in 1994.

Accelerations and decelerations in the decline in Australian smoking prevalence has been noted to be associated with the level of tobacco control advocacy, legislative activity, taxation (and so the price of cigarettes), and national expenditure on social marketing and other tobacco control activities--with most of 1990s being a period of low tobacco control activity and slower falls in smoking prevalence [16,23,24]. It is not possible with only three surveys to make similar claims about the association between the rates of decline in Indigenous smoking prevalences, in either remote or non-remote regions, and the level of total and specifically targeted Indigenous tobacco control activity.

Australian Indigenous smoking prevalences have also not been resiliently static in remote regions, where one quarter of the Indigenous population lives [4]. The declining male smoking prevalence from very high levels and the rise of female smoking prevalence to a lower peak can be explained neatly by the typical characteristics of the stages and shape of the national tobacco epidemics in men and women. Lopez and colleagues describe male smoking prevalence rising and then falling first, with female smoking prevalence rising more slowly, reaching a lower peak then initially falling more slowly than male prevalence [25]. The remote and non-remote Indigenous smoking trends suggest that remote Indigenous Australia is just at an earlier point in the tobacco epidemic than non-remote Indigenous people, plausibly reflecting later access to commercial cigarettes and later and less exposure to tobacco control activities. Sadly, this typical pattern of the tobacco epidemic, and the lag between peaks in smoking prevalence and mortality, predicts that smoking-attributable Indigenous deaths, at the very least amongst remote women, will continue to rise for some years, regardless of any increased tobacco control activities. Many Indigenous premature deaths could have been averted if Indigenous people had been exposed to more intensive tobacco control activities much earlier in the Indigenous smoking epidemic. The reasons for inadequate Indigenous exposure to tobacco control activity may just be the same as the reasons for less Indigenous access to other health services, but may also include the relative neglect of tobacco control compared to other Indigenous health priorities.

The remote and non-remote classifications conflate considerable heterogeneity in smoking prevalence. For example, all of the NT except its capital city Darwin and its immediate environs is classified as remote, yet there is a more than ten-fold difference in lung cancer incidence between its East Arnhem and Alice Springs Rural regions [26], reflecting dramatically different smoking prevalences two decades earlier [27]. All remote (or non-remote) regions are unlikely to have the same smoking prevalence or be at the same point in the tobacco epidemic.

It is difficult to neatly interpret the different Indigenous smoking trends in the different Australian states and territories. Firstly, and most importantly, interpretation is hampered by the smaller subgroup sample sizes and consequently large confidence intervals. Secondly, jurisdictions have different mixes, which cannot be neatly unscrambled, of two factors that could influence Indigenous smoking trends: the proportions of the Indigenous population who live in remote and non-remote areas (and so who are at different stages of the tobacco epidemic) and the amount of generic and targeted Indigenous tobacco control activity.

The main limitation of this study is that almost all differences were not statistically significant; however, some clear patterns emerged. Only by including questions about smoking in the five-yearly Australian Census could these concerns about statistical power be completely addressed. Larger regular national Indigenous surveys are probably impractical: even the smallest of these three surveys interviewed 1 in 45 of the total Australian Indigenous population and took a year to complete interviews [3]. Smokers may have responded differently to the different smoking questions, with their different categories in the three surveys. Some smokers, especially those smoking less than daily, may not have said they were smokers in response to the single question in 1994 [28]. This would mean we have slightly under-estimated the falls in Indigenous smoking.

Nevertheless, it should be possible with consistent smoking questions in new national Indigenous surveys, which are now scheduled to occur every three years, to slowly build an increasingly precise picture of the trends in Australian Indigenous smoking prevalence. More thorough analyses of trends would be possible if ABS provided the data with the same number of replicate weights for each national Indigenous survey so that results could be properly compared and combined. This monitoring should form an essential part of recently accelerated Australian efforts in Indigenous tobacco control [29].

## Conclusion

This study has implications for what tobacco control activities need to be included in future efforts to reduce Indigenous smoking. In the past, the apparent immobility of Indigenous smoking prevalence, whilst total Australian smoking prevalence was successfully falling, could be cited as justification to entirely re-think and re-fashion tobacco control for this Indigenous context [30]. Whilst not denying that every population and setting is different and that tobacco control, like other health promotion, should be sensitive to and acknowledge the local context, we should no longer say that Australian Indigenous smoking is so different that we need to abandon all the strategies that have been proven so effective in the rest of the Australian population and elsewhere. There can be space to trial innovative ideas but the emphasis should be on established tobacco control activities, which should be evaluated in the different local Indigenous settings, and which can readily be made consistent with the key principles for Indigenous tobacco control that were proposed during consultations with Indigenous groups [31]. Indigenous tobacco control need not be considered exceptional, nor should reducing Indigenous smoking be considered exceptionally difficult. However, this study should not be cause for any self-congratulation in Indigenous tobacco control: very high Indigenous smoking prevalences have caused too many premature deaths that could have been prevented by additional and better tobacco control activity.

The implications of this study extend to tobacco control in groups with high smoking prevalence in other countries. First, there is the warning to read closely the published series of reports of national omnibus surveys; in different years, reports may use different categories of smoking status or different age cut-offs and may not always focus on the different smoking prevalences of men and women. More importantly, we should not immediately assume that groups with high smoking prevalence are resistant to the tobacco control activities that are known to be most effective. Such groups may be similarly responsive, but just starting from higher smoking prevalences as they are at an earlier stage in the epidemic.

In Australia, mass media led campaigns have led to falls of similar magnitude in the smoking prevalence in disadvantaged groups (with high smoking prevalence) and less disadvantaged socio-economic groups [32]. An international review of the impact of population-based tobacco control activities on social inequalities in smoking, found overall that tobacco control activities have a similar impact on disadvantaged and less disadvantaged socio-economic groups, but that increased tobacco taxation has a greater impact on more disadvantaged groups [33]. Concerns about the possible lesser impact of smokefree environments legislation on the most disadvantaged may lessen as this legislation expands to more of the public and private spaces used by disadvantaged workers and unemployed people. This is an example of increasing the exposure and access of the most disadvantaged groups to proven tobacco control strategies, rather concentrating than re-inventing new strategies for these groups. In New Zealand, Wilson and colleagues have shown how such enhanced tobacco control has the potential to reduce the mortality gap between Mâori (the indigenous people of New Zealand) and those of New Zealand European ethnicity [34]. Increasing the exposure of Indigenous Australians to proven tobacco control strategies also has the potential to 'Close the Gap' in Australia.

## Competing interests

The author declares that they have no competing interests.
